# Relation among Aromatase P450 and Tumoral Growth in Human Prolactinomas

**DOI:** 10.3390/ijms18112299

**Published:** 2017-11-01

**Authors:** María José García-Barrado, Enrique J. Blanco, María Carmen Iglesias-Osma, Marta Carretero-Hernández, Leonardo Catalano-Iniesta, Virginia Sanchez-Robledo, Manuel Carretero, Julio Joaquín Herrero, Sixto Carrero, José Carretero

**Affiliations:** 1 Department of Physiology and Pharmacology, Faculty of Medicine, University of Salamanca, 37007 Salamanca, Spain; mcio@usal.es (M.C.I.-O.); robledo@usal.es (V.S.-R.); 2 Laboratory of Neuroendocrinology, INCyL and IBSAL, University of Salamanca, 37007 Salamanca, Spain; ejbb@usal.es (E.J.B.); leonardo.catalano@usal.es (L.C.-I.); jcar@usal.es (J.C.); 3 Department of Human Anatomy and Histology, Faculty of Medicine, University of Salamanca, 37007 Salamanca, Spain; martataes@gmail.com; 4 Faculty of Human and Social Sciences, Pontifical University of Salamanca, 37002 Salamanca, Spain; mcarreterogo@upsa.es; 5 Department of Surgery, Faculty of Medicine, University of Salamanca, 37007 Salamanca, Spain; jhpayo@usal.es (J.J.H.); scarrero55@gmail.com (S.C.)

**Keywords:** pituitary gland, aromatase, prolactinoma

## Abstract

The pituitary gland is part of hypothalamic-pituitary–gonadal axis, which controls development, reproduction, and aging in humans and animals. In addition, the pituitary gland is regulated mainly by hormones and neurotransmitters released from the hypothalamus and by systemic hormones secreted by target glands. Aromatase P450, the enzyme responsible for the catabolization of aromatizable androgens to estrogens, is expressed in different parts of body, including the pituitary gland. Moreover, aromatase P450 is involved in sexual dimorphism where alteration in the level of aromatase can initiate a number of diseases in both genders. On the other hand, the direct actions of estrogens, mainly estradiol, are well known for stimulating prolactin release. Numerous studies have shown that changes in the levels of estrogens, among other factors, have been implicated in the genesis and development of prolactinoma. The pituitary gland can produce estradiol locally in several types of endocrine cells, and it is possible that aromatase could be responsible for the maintenance of the population of lactotroph cells and the modulation of the action of central or peripheral regulators. Aromatase overexpression due to inappropriate gene regulation has clinical effects such as the pathogenesis of prolactinomas. The present study reports on the synthesis of pituitary aromatase, its regulation by gonadal steroids, and the physiological roles of aromatase on pituitary endocrine cells. The involvement of aromatase in the pathogenesis of pituitary tumors, mainly prolactinomas, through the auto-paracrine production of estradiol is reviewed.

## 1. Introduction

Aromatase P450 is a complex protein belonging to family 19 of the P450 superfamily of enzymes, termed CYP19. It is found throughout the phylum vertebrates and formed by two components, cytochrome P450 and nicotinamide adenine dinucleotide phosphate (NADPH) cytochrome P450 reductase, located in the membranes of the endoplasmic reticulum; although the human gene is unique compared to the rest of the other members of this superfamily [1,2]. In mammalian systems, Cyp19 uses C19 androgens as their substrate and enzymatically removes C19 to form a phenolic A-ring in the steroid. Depending on which androgens are attached to the prosthetic groups, the products of androstenedione, testosterone, or 16-OH-androstenedione, are estrone, estradiol, or estriol, respectively. The estrogens formed inside the cell interact with estrogen receptors, triggering alterations in gene expression and in cellular functions [2,3,4,5]. Hence, steroids are implicated in many biological processes including development, hypothalamic programming, sexual differentiation, reproductive physiology, behavior, osmoregulation, metabolism, regulation of the hypothalamic–pituitary–gonadal axis, and hypothalamic–pituitary–adrenal axis [6].

Aromatase is extremely critical as an estrogen biosynthetic enzyme. Physiologically, it participates in functions such as glucose homeostasis, lipid homeostasis, brain function, follicular growth, bone mineralization, epiphyseal closure, and the coordination of the ovulatory process [7], and has also been found to be directly responsible for sexual dimorphism in the nervous system. Its expression and activity varies in different parts of the body, and is highly active near gonads, adipose tissues, skin, bone, brain, adrenal gland, liver, placenta, breasts, and hair follicles. It is known to be involved in tumorigenesis and alterations in the level of aromatase can initiate a number of diseases in both genders [2,8,9,10,11,12].

The conversion of androgen to estrogen by aromatase has two effects: on one hand it produces an estrogen molecule and on the other it removes the androgen molecule. Although the amount of estrogens synthesized by this way is quantitatively small, even less than 1%, it should be emphasized that in terms of potency the hormonal activity of estrogens can be up to 1000 times greater that of the androgens [1]. The amino acid sequence of aromatase P450 was described by Hickey and co-workers [13]. This sequence results from the transcription and later translation of the aromatase CYP 19 gene. Different isoforms of aromatase P450 have been reported [14]. These partially differ in the carboxy-terminal amino acid sequence, which could account for some of the differences observed by different authors in the tissue distribution of the enzyme.

Pituitary adenomas are a diverse group of tumors arising from adenohypophyseal cells in the pituitary gland. The proportion of adenomas associated with hormonal hypersecretion or showing further progress is currently unknown. In general, the data on the prevalence of pituitary adenomas are based on anatomical studies with results extracted from either serial autopsies or from magnetic resonance imaging and suggest that the overall estimated prevalence is of 16.7% of all brain tumors [15]. However, local surveys published in Belgium [16] and the UK [17] propose that the prevalence of pituitary adenomas has risen fourfold over the last decades as detected by the better screening of these populations. Although they are considered benign, pituitary adenomas are the cause of significant morbidity due to increased hormone secretion and to a possible compression of neighboring structures [18,19,20,21].

Prolactin-secreting pituitary tumors are called prolactinomas and are highly prevalent along with non-functioning pituitary tumors [22,23]. Their frequency varies with age and sex, being more common in 20- and 50-year-old women than in men; however, later on the frequency of prolactinomas is similar in both sexes [24]. One possible explanation for the increased prevalence of prolactinomas in women may be related to the fact that clinical presentation in women is more evident, usually the classical amenorrhea-galactorrhea syndrome, whereas men may ignore the symptoms of impotence and decreased libido and the diagnosis is often made when signs of compression due to the tumor develop [22].

Additionally, some patients with acromegaly are carriers of mixed adenomas, secreting growth hormone and prolactin [25]. The initiation, development, and progression of adenomas is not well known. However, many factors may influence the proliferation of prolactinomas, such as angiogenesis, apoptosis, growth factors, oncogenes, tumor suppressor genes, and hormone receptors [26]. Studies in animals and humans have demonstrated that estrogen stimulates pituitary tumor transforming gene (PTTG) expression [27]. This gene, the first proto-oncogene to be identified, is weakly expressed in normal tissues. However, it is widely detected in malignant cell lines and in most pituitary tumors [28,29]; thus, estrogens are one of the factors involved in the pathogenesis of prolactinoma.

Recently, after a systematic review, a large number of candidate genes thought to contribute to tumorigenesis, invasion, recurrence, and hormonal hypersecretion in prolactinomas have been revealed [23]. Of the over-expressed genes identified, HMGA2, HST, and SNAP25 showed a clear association with prolactin hypersecretion and tumor formation. The under-expressed genes UGT2B7, Let7, and miR-493 are primarily involved with steroid metabolism and cell cycle regulation, which may contribute directly to the formation and progression of prolactinomas [23].

## 2. Aromatase Expression in Pituitary Gland

The pituitary is an endocrine gland that is affected by the secretion of gonadal steroids and is involved in sexual differences that appear in life. The possibility that aromatase might be produced in this organ and might exert its action at a local level is of particular relevance to this study.

In previous work deadling, with humans and rodents with normal or tumoral pituitaries, we have described the immunohistochemical expression of aromatase in the pituitary gland [8,30,31]. Similar findings were confirmed later on in animal species and different brain structures [32,33,34,35,36,37]. The role played by the transformation of testosterone into estradiol with respect to the secretion of the gland, remains to be fully elucidated. Although it could be debated whether the observations reported in different studies might be developed in the hypothalamus, in the pituitary gland or in both at the same time, such as the fact that the administration of aromatase inhibitors increases circulating luteinizing hormone (LH) levels [38], there is evidence that suggests pituitary aromatase has a potential physiological or pathophysiological importance [9].

We have demonstrated [31], by means of immunohistochemistry, that aromatase is expressed in the rat pituitary gland as early as Day 17 of prenatal development, the cells positive for the enzyme being more prominent and numerous at Day 19 up to birth. Postnatally, towards puberty (around 21 days), differences between males and females begin to appear. Thus, the immunohistochemical expression of aromatase P450 in the adult rat pituitary gland is sexually dimorphic. Finally, non-tumoral pituitary from male and female aged rats hardly express the enzyme [39]. By means of immunohistochemistry, Western blotting, and in situ hybridization, it has been demonstrated that gonadal steroids play an important role in the expression of aromatase in the pituitary gland of adult rats. Moreover, treatment with aromatase inhibitors induces morphometric alterations and changes in the cellular proliferation of some glandular pituitary cells and similar changes can be observed in lactotroph cells or LH-positive gonadotroph cells in knock-out mice for aromatase [40,41].

Although the above findings are suggestive of a defined role for pituitary aromatase, the immunohistochemical expression of the enzyme might not be related to a biological action at the pituitary level. However, the observation of the strong correlation of immunocytochemical expression in the same glandular cell for aromatase and estrogen-receptor α suggests that the enzyme would exert a pituitary auto-paracrine effect, as is discussed in the review of Carretero and co-workers [42].

## 3. Estrogens, Prolactin and Aromatase

The relationship between estrogens, in particular estradiol, and prolactin have been well known for some time. The direct actions of estrogens stimulating prolactin release are well documented in the literature [43,44,45]. In fact, estradiol is an important regulator of prolactin synthesis [46]. The pituitary lactotroph cells have an estrogenic receptor (ER), and there are estrogen-responsive cells [47,48]. Estrogens regulate transcription of the rat prolactin (PRL) gene in vivo [49] through at least two independent mechanisms [50,51] that culminate with an increase in prolactin mRNA levels [46] and that upregulate genes such as vascular endothelial growth factor (VEGF), transforming growth factor β (TGFβ), and galanin [49,50,51,52]. Estrogen stimulate the pituitary vasoactive intestinal polypeptide (VIP)-producing cells and it is a peptide that, in an auto-paracrine way, stimulates prolactin and lactotroph cells [53].

The estrogen-treated rats are an interesting and well-studied model of pituitary hyperplasia. The chronic treatment with estradiol elicits, in a first phase, clear signs of hyperactivity and hyperplasia in lactotroph cells [54,55], hyperprolactinemia and reduction dopaminergic action at the pituitary level. Ovariectomized and ERα-knockout animals have a significant reduction in pituitary PRL levels and the number of lactotrophs cells, suggesting a requirement of estrogen for normal lactotroph function [52]. After the administration of estradiol to adult male rats, lactotroph cells acquire secretory properties and morphological characteristics similar to those found in females, with larger cells that have hypertrophy of the Golgi apparatus, rough endoplasmic reticulum including Nebenkern images, and increases in exocitosis [56,57]. Moreover, estrogens modulate the transdifferentiation of pituitary prolactin and GH cells [58]. In addition, other factors may be involved in these processes, such as interleukins [30,59], dopamine [60,61], and thyrotropin-releasing hormone (TRH) [62,63].

The genomic and non-genomic effects of estrogens have also been reported, using rat pituitary-derived cell lines such as GH3 [64,65]. The GH3 cell line, one of the models developed to study prolactinomas in vitro, was generated by treating a rat with high doses of estrogens, in which a prolactinoma developed [6].

Estradiol is known to rapidly activate many signaling molecules, including insulin-like growth factor 1 receptor (IGF-IR), epidermal growth factor receptor (EGFR), and mitogen-activated protein kinase (MAPK) in breast cancer cells. Blockade of estradiol synthesis with aromatase inhibitors or antagonism of its action with anti-estrogens represents first-line treatments for patients with estrogen-receptor-positive breast cancer [66].

The epidermal growth factor receptor (EGFR, ErbB, and HER) family comprises four subtypes of families that are associated with transmembrane tyrosine kinase receptors. EGFR and HER2 are expressed in normal anterior pituitary cells, including lactotrophs cells and induce prolactin release. Moreover, EGFR/HER2 signaling regulates tumor growth and hormone production in lactotroph tumors among others [67]. In EGFR- and HER2-overexpression transgenic models larger tumors appear that respond to tyrosine kinase inhibitors [68]. Although the relation among aromatase, estrogens, and EGFR have been studied in different tumors such as breast [69], endometrial [70], lung [71], and liver [72] tumors, among others, the relationship between EGFR and aromatase in pituitary gland or prolactinomas has not been well analyzed to date, but its participation should not be ruled out.

Pituitary aromatase is produced in different cell types, including normal and tumoral lactotroph cells [8,35]. This is of particular relevance since the transformation of aromatizable androgens into estradiol means that, in males the androgenic inhibitory effect on prolactin is transformed, locally and/or intracellularly, into a stimulatory effect, allowing the population of lactotroph cells to be high in this sex. The treatment of male rats with aromatase inhibitors elicits a decrease in the activity and proliferation of lactotroph cells [41], and similar findings can be observed in aromatase knock out (KO) mice [73].

## 4. Possible Involvement of Aromatase P450 in the Pathogenesis of Prolactinomas

**Figure 1:**
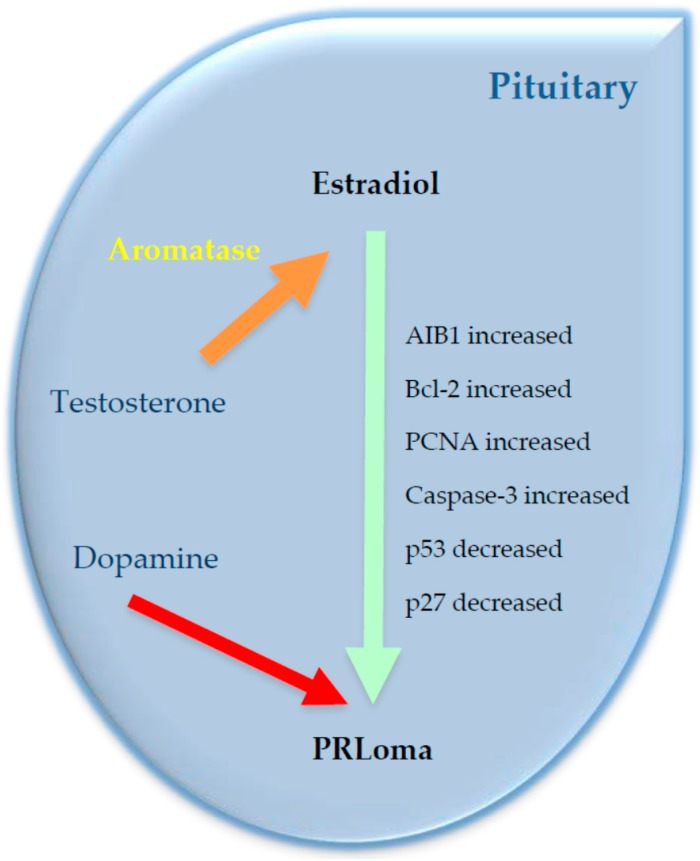
Schematic diagram of the relation among aromatase P450 and growth markers in prolactinomas. Dopamine and steroids are regulators of prolactin production. Dopamine suppresses the prolactin release (red arrow: inhibition), while estradiol is an important regulator of prolactin synthesis (blue arrow: stimulation). The conversion of testosterone to estradiol is mediated by aromatase (orange arrow: stimulation). When some markers present inadequate regulation or their intracellular localization altered, they can lead to an overexpression of aromatase and trigger prolactinomas. In prolactinoma, AIB1, Bcl-2, and proliferating cell nuclear antigen (PCNA) markers are increased and Caspase-3 p53 and p27 are decreased (Modified of [73]).

As described above, estrogen synthesis is a process that does not necessarily derive from peripheral sources but can be synthesized de novo in different tissues [74] from testosterone by the action of aromatase [1,2,41]. Chronic treatment with estradiol induces, in a first phase, cell hyperplasia prolactin, constituting, in a second phase, prolactinoma, depending on the duration of treatment [75]. In the same manner, numerous studies have shown that supraphysiological levels of estrogens, among other factors, have been implicated in the genesis and development of prolactinomas and VIPomas [25,27,29,76,77]. Thus, if one of the factors that has been implicated in the genesis of prolactinomas is the estradiol, because the tumor develops following chronic treatment with the steroid, and the pituitary produces estradiol by acting of aromatase, it is not unreasonable to consider the possibility that the development of pituitary tumors, and in particular the development of prolactinomas, could be related to the local production of estradiol from testosterone through the action of aromatase (see Figure 1).

Although mutations or polymorphisms of the CYP19 have been described and their participation on the regulation of postmenopausal circulating sex hormones [78] or in the development and prognosis of breast cancer [79], there is no evidence of genetic variations and the regulation of pituitary prolactin secretion or the development and growth of prolactinomas.

In our laboratory, evidence has been found suggesting an important relationship between the pathogenesis of prolactinomas and the overexpression of aromatase in the pituitary gland of rodents and humans. For this reason, two series, one of 105 adenomas obtained from female Sprague–Dawley rats of 24 months of age [10] and the other of 87 spontaneous adenomas from women between 23 and 67 years old obtained from surgical treatment were analyzed [42]. In both cases, immunoreactivity to the enzyme appeared in endothelial cells and glandular cells. Akinci et al. [80] have detected the presence of aromatase levels higher in patients with prolactinoma than normal pituitary tissues. Although no difference was found between men and women, aromatase expression was shown to be higher in men with an invasive adenoma than in those without invasive adenoma. Similarly, the association among aromatase and pituitary tumors has been described in both, men and women [12,81]. The importance of the involvement of aromatase in the development of prolactinomas is seen upon observing that mice KO for aromatase do not develop prolactinomas [73].

Contrary to what occurs for breast tumors, there are only a few studies analyzing the clinical relevance of aromatase in prolactinomas, and the use of aromatase inhibitors is useful in the restoration of gonadal function by testosterone in men with prolactinomas [81,82,83,84].

## 5. Balance among Cell Proliferation and Apoptosis in Prolactinomas

The maintenance of tissue homeostasis in the anterior pituitary gland results from a balance between cell proliferation and death by mechanisms that are tightly regulated. Classically, the proliferative cell fraction in the anterior pituitary of adult animals is described as low, while it is high during development [85,86]. The effects of estrogens on the pituitary gland are not only those that are classically described as mitogenic, but anti-proliferative and pro-apoptotic actions are also apparent [87].

After extensive studies were carried out regarding changes in proliferation and apoptosis in pituitary tumors, higher apoptotic activity pituitary carcinomas compared with adenomas was observed, indicating that apoptosis could be a useful prognostic marker [88]. However, other authors found that apoptotic indices were not predictive of the growth rate of non-functioning pituitary tumors [89,90]. These discrepancies may be due to the technique used to detect apoptotic cells in each study. Kontogoergos et al. [88] showed that functioning adenomas had higher indices than did non-functioning tumors, although the highest apoptotic indices were observed in corticotrope adenomas, and in untreated adenomas, particularly prolactinomas.

The expression of Bcl2 and Bcl-2-like protein 4 (BAX), respectively, has been used as anti-apoptotic and pro-apoptotic factors. In non-functioning adenomas as well as in PRL-secreting adenomas the expression of Bcl-2 found decreased [80] and BAX protein was increased when associated with pituitary tumor progression. In female rats, estrogens induce changes in the balance of pro- and antiapoptotic Bcl-2 family proteins (Bcl-2 and BAX) and inhibition of the nuclear factor kappa-light-chain-enhancer of activated B cells (NFκB) pathway [91,92]. In the male, 17β-estradiol exerts rapid apoptotic action in lactotropes, somatotropes, or gonadotropes cells of pituitary gland. However, the effects of gonadal steroids on the expression of Bcl-2 and BAX, in the anterior pituitary gland of male rats, have not been modified [93].

Previous findings from our laboratory show that the expression of pituitary aromatase was higher in male than in female rats [31], and aromatase expression in lactotropes is negatively correlated with age and almost completely disappears in the pituitary gland of aged male rats [39]. Therefore, aromatase can locally generate high levels of estradiol that can act through auto-paracrine mechanisms [7,36]. The in vitro and in vivo expression of aromatase is in the lactotroph cell of male rats and is involved in the control of proliferation and prolactin release by transforming testosterone to estradiol [41]. Moreover, the pituitary aromatase activity could be involved in the regulation of the apoptosis of pituitary cells [93]. Other studies have shown that, in patients with acromegaly and prolactinoma, aromatase was high, was negatively correlated with Ki-67 score, and was higher in the pituitary of patients with complete postoperative remission than without remission [12].

## 6. Estrogenic Receptors, Prolactinomas, and AIB1

The biological effects of estrogens are mediated by their two nuclear receptors—estrogenic receptor α (ERα) and estrogenic receptor β (ERβ)—both of which are necessary for hormone action in target tissues [94]. They play an important mitogenic role, stimulating the proliferation of lactotroph cells [29,95], acting directly on these cells. The presence of estrogen receptors in the pituitary gland has been described in adult rats of both sexes as well as the direct action of estradiol on pituitary cells [51,56,96]. The expression of ERα and ERβ are also present in human pituitary adenomas [48,97,98]. However, in male rats, the expression of ERα is very high compared with ERβ in the anterior pituitary [94].

In animals and human studies, estrogens induce the expression of pituitary tumor transforming gene (PTTG), a proto-oncogene that regulates cell cycle progression, proliferation, differentiation, repair, transformation, and angiogenesis [99]. This proto-oncogene is weakly expressed in normal tissues; however, it is widely detected in malignant cell lines and in most pituitary tumors, including prolactinomas [100,101]. Likewise, PTTG stimulates fibroblast growth factor 2 (FGF2) and vascular endothelial growth factor (VEGF) production, accelerates tissue angiogenesis, and facilitates pituitary tumor progression through local invasion of the surrounding tissues [99,102]. On the other hand, AIB1, an important factor in the development of breast cancer [103], is overexpressed by the action of 17-β-estradiol [104], and AIB1 may be an important diagnostic and therapeutic target in breast cancer [105]. The relation between ERα and AIB1 is well established [103,106,107], and the over-expression of AIB1 could be related to the increases in the incidence of pituitary tumors in mice [108].

Previous findings from our laboratory demonstrate that the overexpression of aromatase in human prolactinomas was associated with the presence of the ERα and the overexpression of the estrogenic mitogen coactivator AIB1 [30] and could be related to proliferative or anti-apoptotic roles of AIB1 as has been described for others tumors [108,109,110,111,112,113]. One of the most interesting results observed in these study was the presence of a different, cytoplasmic or/and nuclear, subcellular distribution of AIB1: 17% showed reaction only in the cytoplasm, 24% presented it only in the nucleus, and 59% had both cytoplasmic and nuclear reactions. As a translational coactivator, AIB1 develops its action in the cell nucleus. In fact, in quiescent mammary epithelial cells or cell cultures devoid of growth factors, it is located in the cell cytoplasm [96]; however, when the nuclear localization predominates, cells are proliferated [98] in a manner similar to that in which the development of breast tumors occurs [114].

In cell lines, AIB1 can be observed in different subcellular compartments, cytoplasmic or nuclear [115,116], a fact has been linked with its degradation. Cytoplasmic AIB1 has a half-life longer than nuclear AIB1, suggesting the existence of cytoplasmic lysosomal degradation, although less important than nuclear degradation, which is carried out by the MG132 proteasome [115]. Intranuclear localization of AIB1 could be related to the activation of estrogen receptors, whereas cytoplasmic localization AIB1 could be linked to an increase in half-life and activation AP-1. In the case of the pituitary gland and in particular the prolactin cells, AP-1 is one of the most important mechanisms in the activation of these cells [117]. Therefore, the nuclear localization of AIB1 in prolactinomas is associated with the cellular proliferative status, whereas cytoplasmic-AIB1 is related to apoptosis [30].

## 7. P53 and P27 Proteins in Prolactinomas

The p53 protein is a nuclear phosphoprotein that acts as a tumor suppressor by inhibiting cell cycle progression and the phosphorylation of retinoblastoma protein [118]. Its function is altered, generally, by point mutations in the gene encoding it in over 50% of human cancers [119]. The relationship between estrogen receptor and p53 has already been corroborated [120,121,122,123]. It is suggested that ERα directly interacts with p53, and this association could prevent the p53-mediated apoptotic response [122]. ERα antagonizes the pro-apoptotic function of p53, promoting cancer cell survival. Recently, in breast cancer cells, it has been shown that ERβ physically interacts with p53, reduces ERα-p53 binding, antagonizes ERα-p53-mediated transcriptional regulation, and could generate changes epigenetic in histone methylation [121]. P53 also inhibits the expression of aromatase by binding to a p53 response element on the aromatase promoter to be repressed by prostaglandin E2 (PGE2). It was demonstrated that the loss of p53 leads to the stabilization of hypoxia-inducible factor 1-alpha (HIF1α) and metabolic regulator protein kinase M2 (PKM2) besides stimulates their interaction with the aromatase promoter and induces an increase in aromatase expression and activity [124].

The presence of p53 in pituitary adenomas is well documented in the literature [26,88,125,126,127,128]. The importance of the role that p53 can play in the cytophysiology and the progression of pituitary adenomas has become relevant since DNA damage in cells from pituitary tumor lines induces the cell cycle arrest mediated by p53 and determines whether or not reparable damage occurs before continuing with cell division [129,130]. Therefore, mutation, deletion, or p53 inactivation clearly favors tumor progression.

There may be some relationship between factors involved in the development of prolactinomas and the role played by p53 and Bcl-2. In the pituitary gland, the pituitary apoptosis associated with increased p53 appears only at very low levels or the absence of estrogen [131]. On the other hand, the apoptotic activity in the pituitary adenomas treated with bromocriptine depends on activation of p53 and suppression of Bcl-2 [132]. Despite the fact that p53 expression is reported to be associated with the tumor invasiveness in pituitary tumors, there are few reports on the role of p53-dependent apoptosis in pituitary tumor therapy [133]. Additionally, the presence of p53 in invasive or large human pituitary adenomas [26,127] is controversial. However, it has been established that the expression of p53 is very important when assessing the prediction of the behavior that follows human pituitary adenomas [88,134], with a higher expression in recurrent adenomas compared to those that are non-recurring [135] and adenomas bromocriptine-resistant [100]. In our laboratory, we found that around 76% of prolactinomas were positive for p53, similar to results described previously by other laboratories [125]. However, there are few studies analyzing the percentage of p53-positive pituitary adenomas and, in general, the few studies that analyze this data refer mainly to pituitary adenomas in general or pituitary tumor lines instead of prolactinomas in particular [26].

There are no descriptions relating to the intracellular localization of p53 with other biological actions. Given that p53 is a protein that carries out its action in the nucleus, the arrest of p53 in the cytoplasm of tumor cells could avoid its biological action on DNA binding to promoters that regulate the cell cycle and apoptosis. We have seen that the pattern of reaction to p53 and in particular, the intracellular localization of the reaction varies greatly from one tumor to another. Although 76% of prolactinomas showed positive p53 cells, 58% of p53-positive prolactinomas showed some isolated p53-positive cells. It is very important to consider that in the prolactinomas that showed many positive p53 cells, the protein was arrested in the cytoplasm, and, although some cells could have a nuclear reaction these cells were very low, always below 0.6% of the positive cells.

In pituitary tumor cell lines, bromocriptine-resistant p53 adopts a mutant conformation that precludes its nuclear translocation and transcriptional activity [136]. However, mutations of p53 in pituitary benign adenomas have not been described [26,137]. In most of the p53-positive human prolactinomas, the protein is located in the cytoplasm of the cell and the intracellular localization of the protein could be very important in the growth of the tumor. In addition, AIB1 and p53 could be related to in the pathogenesis of prolactinomas because, in HeLa cells, p53 interacts with specific “rub” regions of AIB1, and AIB1 can modulate p53 transactivation [138]. This is similar to what occurs in other tumors, such as the correlation between the overexpression of AIB1 and p53 positivity in breast cancer [106] or in colorectal cancer [139].

P27/(KIP1) is a cyclin-dependent kinase inhibitor that plays important roles in the regulation of cell-cycle progression [134]. An increase in levels of p27/KIP1 protein typically causes cells to arrest in the G1 phase of the cell cycle [140]. In different types of tumors, it has been revealed that growth factors present outside epithelial cells, such as transforming growth factor beta (TFGβ), arouse p27 levels inside a cell [133]. Several studies found significantly lower p27/KIP1 levels in non-functioning adenomas [141], although other research has exhibited higher proliferation rates in these tumors [142]. There is evidence that p27 protein expression decreases during development and progression in pituitary adenomas, including prolactinomas, compared with the normal gland [90,133,143]. Recurrent adenomas and malignant tumors showed a p27/KIP1 expression that was lower than non-recurrent adenomas. However, different studies have failed to detect any mutations within the p27 gen [144]. The significance and mechanisms underlying reduced p27/KIP1 levels in pituitary tumors is uncertain.

Recently, Martins and co-workers [145] have confirmed p27 underexpression in pituitary adenomas and have thus provided further evidence of the involvement of the post-translational machinery, although this phenomenon cannot be explained either by the mis-expression of p27 translational regulators DKC1, RPS13, miR221, and miR222 or by DKC1 mutations directly. For the moment, the participation of p27 in the development and evolution of prolactinomas is unclear and requires further research.

## 8. Conclusions

Because aromatase is synthesized in the pituitary gland, it has been able to produce estradiol locally in several types of endocrine cells. Our previous results suggest that aromatase is an auto-paracrine regulatory factor by means of estrogens for the maintenance of the population of lactotroph cells and that it modulates the action of central or peripheral regulators. Moreover, its overexpression is present in prolactinomas. Estrogens are involved in the regulation of the proliferation and apoptosis, which play an important role in the maintenance of pituitary cell populations, and implicated in the pathogeny of anterior pituitary tumors, especially prolactinomas. The clinical relevance for the role of aromatase in the genesis and/or growth of prolactinomas, and the consideration of aromatase as a therapeutic target, mainly in dopaminergic-resistant tumors, are fields that need be explored.

## Abbreviations

NADPH	Nicotinamide adenine dinucleotide phosphate
LH	Luteinizing hormone
PRL	Prolactin
VIP	Vasoactive intestinal polypeptide
TRH	Thyrotropin-releasing hormone
IGF-IR	Insulin-like growth factor 1 receptor
EGFR	Epidermal growth factor receptor
MAPK	Mitogen-activated protein kinase
BAX	Bcl-2-like protein 4
NF-κB	Nuclear factor kappa-light-chain-enhancer of activated B cells
ER	Estrogenic receptor
PTTG	Pituitary tumor transforming gene
VEGF	Vascular endothelial growth factor
AIB1	Amplified in breast 1 protein
PCNA	Proliferating cell nuclear antigen
